# Correction: Tang et al. Vitamin K2 Modulates Mitochondrial Dysfunction Induced by 6-Hydroxydopamine in SH-SY5Y Cells via Mitochondrial Quality-Control Loop. *Nutrients* 2022, *14*, 1504

**DOI:** 10.3390/nu15163540

**Published:** 2023-08-11

**Authors:** Hengfang Tang, Zhiming Zheng, Han Wang, Li Wang, Genhai Zhao, Peng Wang

**Affiliations:** 1 Institute of Intelligent Machines, Hefei Institutes of Physical Science, Chinese Academy of Sciences, Hefei 230031, China; hengfangt@163.com (H.T.); wh_yingwang@163.com (H.W.); liwang@ipp.ac.cn (L.W.); zhgh327@ipp.ac.cn (G.Z.); 2 Science Island Branch of Graduate, University of Science and Technology of China, Hefei 230026, China; 3 Anhui Key Laboratory of Environmental Toxicology and Pollution Control Technology, Hefei Institutes of Physical Science, Chinese Academy of Sciences, Hefei 230031, China; 4 CAS (Hefei) Institute of Technology Innovation Co., Ltd., Hefei 230088, China

## Missing Funding

In the original publication [1], the funding from Anhui Province’s key research and development program, 202004b11020014, to Zhiming Zheng was not included. The full funding should be: 

**Funding:** This work was sponsored by financial support from the Major Projects of Science and Technology of Anhui Province (202103a06020003), Anhui Province’s key research and development program (202004b11020014), China National Key Research and Development Program (2019YFA0904300 and 2019YFA0904304), Key Research and Development Plan of Anhui Province (1804b06020342), Natural Science Foundation of Anhui Province (1908085MB48 and 1908085MB43), and National Natural Science Foundation of China (32070088).

## Text Correction

There was an error in the original publication. The wrong reagent information for antibody Bcl-2 was included. Corrections have been made to the 8th and 9th sentences of Section 2.8:

PINK1, MFN1, MFN2, Nrf2, Bax, TFAM (Bimake, Houston, TX, USA), Bcl-2 (Cell Singling Technology, Boston, MA, USA) and β-actin (Proteintech, Wuhan, China).

There was an error in the original publication. Due to carelessness, there was a word used incorrectly in the sentence. Corrections have been made to the 5th and 6th sentences of Section 3.7.

The results showed that 6-OHDA slightly increased the expression of PINK1 and Parkin proteins, compared to that in the control group.

## Error in Figure

In the original publication, there was a mistake in Figure 2 as published. Since the information of the previously used antibody Bcl-2 was wrong, we purchased a new Bcl-2 antibody and re-performed the relevant experiments, to make replacements for Figure 2D. The corrected version of Figure 2 appears below. 


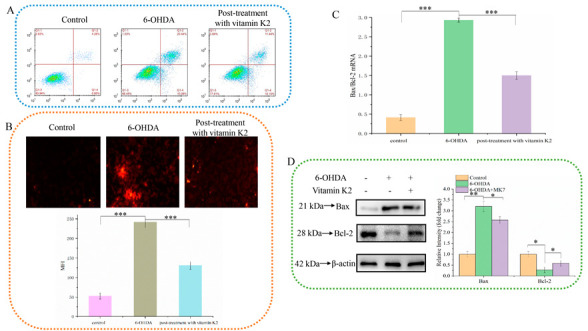


The authors apologize for any inconvenience caused and state that the scientific conclusions are unaffected. This correction was approved by the Academic Editor. The original publication has also been updated.
